# KGG: a fully automated workflow for creating disease-specific knowledge graphs

**DOI:** 10.1093/bioinformatics/btaf383

**Published:** 2025-06-28

**Authors:** Reagon Karki, Yojana Gadiya, Andrea Zaliani, Bishab Pokharel, Negin Sadat Babaiha, Marek Ostaszewski, Martin Hofmann-Apitius, Philip Gribbon

**Affiliations:** Fraunhofer Institute for Translational Medicine and Pharmacology (ITMP), Hamburg, 22525, Germany; Fraunhofer Cluster of Excellence for Immune-Mediated Diseases (CIMD), Frankfurt, 60590, Germany; Fraunhofer Institute for Translational Medicine and Pharmacology (ITMP), Hamburg, 22525, Germany; Fraunhofer Cluster of Excellence for Immune-Mediated Diseases (CIMD), Frankfurt, 60590, Germany; Bonn-Aachen International Center for Information Technology (B-IT), University of Bonn, Bonn, 53113, Germany; Fraunhofer Institute for Translational Medicine and Pharmacology (ITMP), Hamburg, 22525, Germany; Fraunhofer Cluster of Excellence for Immune-Mediated Diseases (CIMD), Frankfurt, 60590, Germany; Fraunhofer Institute for Translational Medicine and Pharmacology (ITMP), Hamburg, 22525, Germany; Fraunhofer Cluster of Excellence for Immune-Mediated Diseases (CIMD), Frankfurt, 60590, Germany; University of Hamburg, Department of Informatics, Hamburg, 22527, Germany; Bonn-Aachen International Center for Information Technology (B-IT), University of Bonn, Bonn, 53113, Germany; Department of Bioinformatics, Fraunhofer Institute for Algorithms and Scientific Computing (SCAI), Sankt Augustin, 53757, Germany; Luxembourg Centre for Systems Biomedicine (LCSB), University of Luxembourg, Belvaux, L-4367, Luxembourg; Bonn-Aachen International Center for Information Technology (B-IT), University of Bonn, Bonn, 53113, Germany; Department of Bioinformatics, Fraunhofer Institute for Algorithms and Scientific Computing (SCAI), Sankt Augustin, 53757, Germany; Fraunhofer Institute for Translational Medicine and Pharmacology (ITMP), Hamburg, 22525, Germany; Fraunhofer Cluster of Excellence for Immune-Mediated Diseases (CIMD), Frankfurt, 60590, Germany

## Abstract

**Motivation:**

Knowledge graphs (KGs) in life sciences have become an important application of systems biology as they delineate complex biological and pathophysiological phenomena. They are composed of biological and chemical entities represented with standard ontologies to comply with Findable, Accessible, Interoperable and Reusable (FAIR) principles. Alongside serving as a graph database, KGs hold the potential to address complex scientific queries and facilitate downstream analyses. However, the process of constructing KGs is expensive and time consuming as it primarily relies on manual curation from published literature and experimental data. The existing text-mining workflows are still in their infancy and fail to achieve the accuracy and reliability of manual curation.

**Results:**

Knowledge graph generator (KGG) is an automated workflow for representing chemotype and phenotype of diseases and medical conditions. It embeds the underlying schema of curated databases such as OpenTargets, Uniprot, ChEMBL, Integrated Interactions Database and GWAS Central resembling a clockwork-esque mechanism. The resultant KG is a comprehensive and rational assembly of disease-associated entities such as proteins, protein-related pathways, biological processes and functions, genetic variants, chemicals, mechanism of actions, assays and adverse effects. As use cases, we have used KGs to identify shared entities for possible link of comorbidity and compared them with KGs from other sources. We have also demonstrated a use case of identifying putative new targets and repurposing drug candidates in Parkinson’s Disease. Lastly, we have developed reusable workflows to explore drug-likeness of chemicals and identify structures of proteins.

**Availability and implementation:**

The resources and codes for KGG are publicly available at: https://github.com/Fraunhofer-ITMP/kgg.

## 1 Introduction

### 1.1 Knowledge graphs and their applications

Knowledge graphs (KGs) are advanced forms of networks that capture the semantics of the constituent entities and the interactions among them. They are becoming increasingly popular due to their ability to efficiently support data warehousing (Wang and Chen 2020, Dibowski and Schmid 2021). In particular, KGs facilitate ontology-driven data consolidation via integration of heterogeneous and multi-modal data and serve as a graph database (Brandizi *et al.* 2018). Importantly, the true essence of KGs lies in their potential to answer complex queries and form the basis of domain-specific analyses. For instance, they have been used in representing complex knowledge of cyber security and deployed in the prediction and traceability of cyber-attacks (Zhang and Liu 2020). Similarly, KGs are also used in the detection of fake news on social media platforms (Mayank *et al.* 2022).

In the context of biomedicine and life sciences, KGs have become one of the most widely used applications of systems biology, mainly because of their innate ability to enable a holistic understanding of biological systems. They delineate disease-associated biological and pathophysiological phenomena by enabling systematic assembly of various inter-related entities such as proteins and their biological processes, molecular functions and pathways, mutations and single nucleotide polymorphisms (SNPs), chemicals and their mechanism of actions and adverse effects. Such a resource in place serves as a basis for various use cases and downstream analyses in healthcare, pharmaceutical, and clinical applications. In this regard, cause-and-effect models for Alzheimer’s Disease (AD), have been built to identify putative dysfunctional mechanisms that manifest AD (Kodamullil *et al.* 2015). Likewise, similar to Gene Set Enrichment Analysis (GSEA) resources such as Molecular Signature Database (MSigDB) and Database for Annotation, Visualization and Integrated Discovery (DAVID), a KG-derived mechanism enrichment platform for neurodegenerative diseases (NDDs) (i.e. NeuroMMSig) has been developed for interpretation of clinical data (Domingo-Fernández *et al.* 2017). Another potential of KG is demonstrated by the work done in COVID-19 PHARMACOME where the authors were able to validate their *in silico* findings under *in vitro* settings (Domingo-Fernández *et al.* 2021). In this work, they firstly created a comprehensive drug-target-mechanism-centric KG and later performed network analysis to predict candidate drug pairs for combinatorial therapy against COVID-19. These candidate drug pairs were tested on virus-induced cytopathic effect in CaCo-2 cells which revealed Remdesivir with Thioguanosine and Nelfinavir with Raloxifene to have the best synergistic effects among 47 combinations.

With the recent advances in machine-learning (ML) and artificial intelligence (AI) methods, KGs have also been a favorable playground for generation of new insights, especially for link or edge predictions between drugs and diseases (Xu and Wang 2013), drug repurposing (Himmelstein *et al.* 2017), drug–drug interactions (Celebi *et al.* 2019), comorbidity risk prediction (da Silva *et al.* 2019), patient diagnosis (Choi *et al.* 2020, Nelson *et al.* 2022), and drug safety (Shang *et al.* 2019). In this line, DREAMwalk tool parses multi-layer semantic knowledge of drugs, genes and diseases from KGs to decipher novel drug-disease associations and facilitate drug repurposing (Bang *et al.* 2023). Another study developed a supervised ML method to perform survival analysis and predict cancer sub-groups of patients using a KG representing multi-omics patient data (Liu *et al.* 2022). Similarly, KGs have also been deployed in improving patient safety by predicting unreported adverse effects of drugs followed by the validation with electronic health records (Bean *et al.* 2017). Tiresias is yet another framework that learns from similarity-based features (i.e. mechanism of action, physiological effect, pathways, side effect, etc.) of drugs to predict drug-drug interaction (Abdelaziz *et al.* 2017).

### 1.2 Existing KG frameworks and pipelines

The advent of systems biology has led to a number of tools and frameworks such as CellDesigner (Funahashi *et al.* 2006), OpenBEL (Slater 2014), BioPAX (Demir *et al.* 2010), and SBML (Hucka *et al.* 2003) for modeling complex biological phenomena. These are life science domain-specific knowledge representation languages used by curating experts to identify, extract and integrate heterogeneous data from various sources such as literature, experiments and biomedical data. However, the manual effort needed for curation is time consuming and expensive. In recent years, text-mining workflows have been deployed to facilitate natural language processing (NLP) by performing tasks such as text parsing, named-entity recognition, text classification, semantic analysis, etc., to eventually accelerate curation tasks. A number of semi-automated [e.g. BELIEF (Madan *et al.* 2015) and BELminer (Ravikumar *et al.* 2017)] and fully automated [i.e. BERE (Hong *et al.* 2020) and Kairntech Sherpa (Geißler 2020)] text-mining workflows have been developed to speed up the process of knowledge extraction. Even more recently, large language models (LLMs), which use deep-learning architecture, have become the hottest AI topic and are revolutionizing the way data are processed. As opposed to the text-mining system’s principle of analyzing textual relationships through aforementioned tasks, LLMs generate text through iterative predictions of words.

The existing text-mining systems, however, have poor recall and precision which is attributed more to the complexity of our human language (i.e. word/phrase ambiguities, words with multiple meanings, language differences, etc.) rather than the underlying algorithm itself (Lee *et al.* 2020, Lan *et al.* 2021, Khurana *et al.* 2023). In this context, it cannot be ruled out that the relatively novel LLMs will have their own caveats and hence need further improvement. In fact, shortcomings such as factually misleading information (i.e. hallucinations), lack of provenance (i.e. source on origin of information) and breach of ethical regulations and data privacy have already been reported (Barman *et al.* 2024, Ong *et al.* 2024). Nevertheless, it will be the case that text-mining systems and LLMs will continue to evolve and to facilitate the knowledge extraction process.

On the other hand, curated databases which contain high-quality data have been operational since decades. According to Database Commons, a total of 6408 databases are currently available. Among them, Kyoto Encyclopedia of Genes and Genomes (KEGG), one of the progenitors of biological databases, was launched in 1995 (Kanehisa *et al.* 2017). It serves as a computer model of biological systems which is built by capturing interactions between biological and chemical entities. Likewise, UniProt was launched in 2002 with the purpose of storing protein sequences along with information about their functions, domain structures, variants, and post-translational modifications (The UniProt Consortium 2023). Databases as such make enormous efforts to manually curate relevant biological knowledge and data from various resources, similar to the protocols of creating KGs but to a much larger scope and extent.

In this work, we focused on leveraging the quality of open and public curated databases, whose data is regarded as gold standard of NLP workflows. Here, we present Knowledge graph generator (KGG), a fully automated workflow to systematically capture and represent chemotype-phenotype of diseases and medical conditions. The workflow embeds underlying schema of curated biological and chemical databases such as Open Targets Platform (OTP) (Ochoa *et al.* 2021), UniProt (The UniProt Consortium 2023), ChEMBL (Zdrazil *et al.* 2024), GWAS Central (Beck *et al.* 2023) and Integrated interactions database (IID) (Kotlyar *et al.* 2016) to fetch relevant information about entities in real-time. The KGG is able to generate KGs with a minimum input, i.e. a disease name prompted by the user to start the entire workflow. Moreover, the users have the possibility to customize the size and content of KG as the workflow is interactive and takes user input to choose the number of proteins and clinical trial phases of chemicals. The final KG is a comprehensive and rational assembly of disease-associated entities including proteins, protein-related pathways, biological processes and functions, chemicals, mechanism of actions, assays and adverse effects, SNPs and mutations. Additionally, we present use cases to identify shared entities between recently suggested comorbidity between COVID-19 and AD followed by comparison of our KGs with KGs from other resources. Moreover, we also contextualize the knowledge of proteins, their druggability state and associated pathways to identify putative new targets and repurposing drug candidates in Parkinson’s Disease (PD). Lastly, we propose methods for mapping entities across databases, identifying Protein Data Bank (PDB) structures of proteins (Burley *et al.* 2023) and exploring drug-likeness of chemicals.

## 2 Materials and methods

### 2.1 KGG workflow

**Figure 1.**
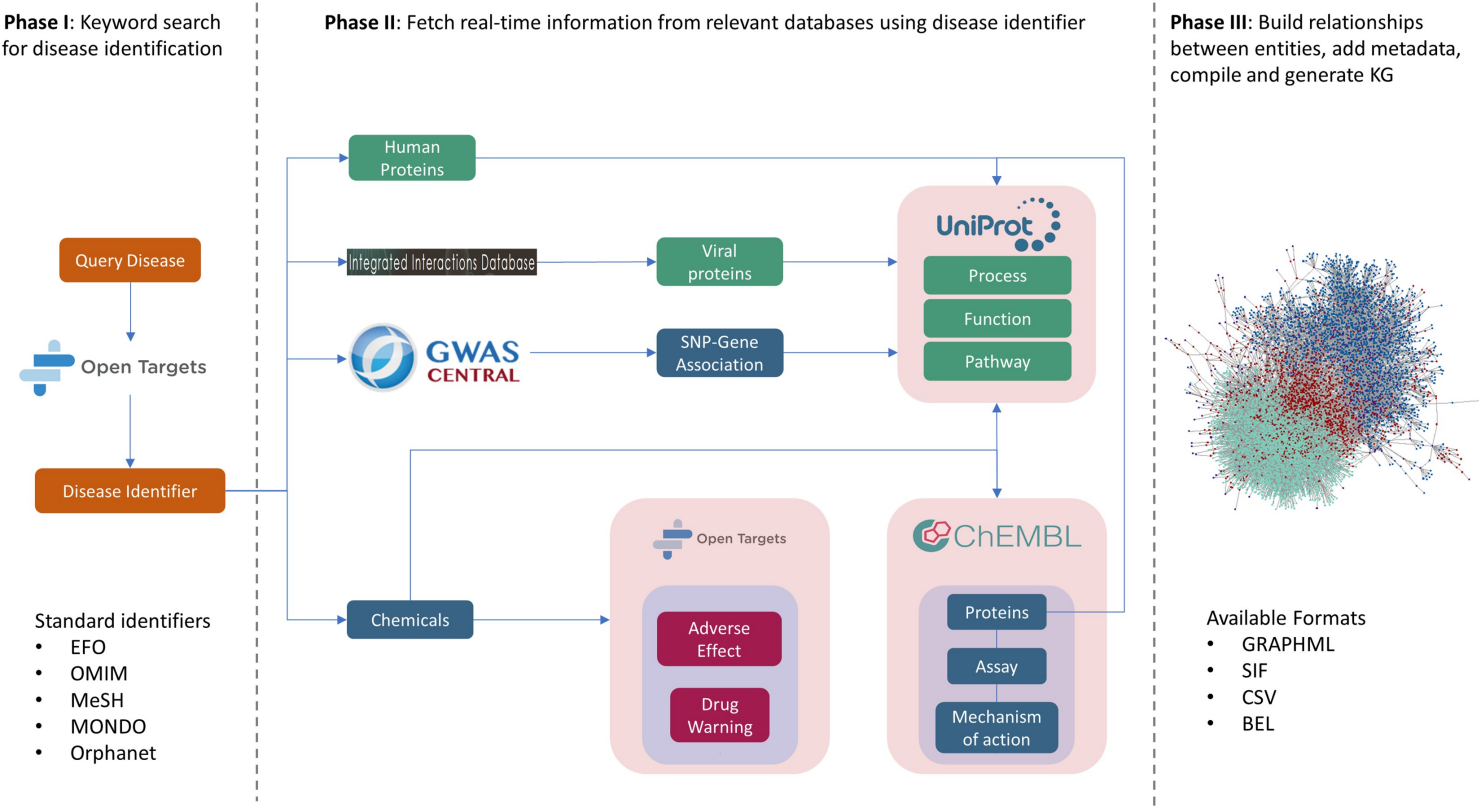
A schematic representation depicting three phases of the KGG workflow.

The programmatic scripts and methods for KGG are written in python (version 3.10) and are available at https://github.com/Fraunhofer-ITMP/kgg. This automated workflow for creating disease-specific KGs is subdivided into three phases and are described below (Fig. 1).

#### 2.1.1 Phase I: disease lookup and identification

The KGG workflow uses standard disease identifiers from widely accepted ontologies such as Experimental Factor Ontology (EFO), Online Mendelian Inheritance in Man (OMIM), Medical Subject Headings (MeSH), Mondo Disease Ontology (MONDO) and so on. Therefore, the identification of a proper disease identifier for a specific disease is the foremost task in the workflow. In order to facilitate this task, we have designed KGG in such a way that the users can search disease names as keywords which are eventually passed as queries to the OTP’s application programming interface (API) (Ochoa *et al.* 2021). This step of the KGG workflow is termed as disease lookup which yields a list of diseases and identifiers closest to the keyword search. The users are then prompted to identify their disease of interest(s) and the process of generating a KG can be initiated by using the corresponding identifier(s). One important aspect worth mentioning about the workflow is that the choice of disease identifier is the most influential step for the KG generation process as it affects the overall size and content of the KG. Reason for this is that the hierarchy of disease concepts represented in the ontologies are used as seeds to create the KG and this will directly affect the retrieval of associated proteins and drugs. For example, if a user is interested to create a KG for Alzheimer disease (AD) (MONDO: 0004975), the KG will represent both its sub-types [i.e. late-onset Alzheimers disease (MONDO: 1001870) and familial Alzheimer disease (FAD) (MONDO: 0100087)], and thereby also the proteins and drugs associated with them. In the case that the user is interested only in FAD, the KG workflow can be fed with its corresponding identifier. A visual representation of this example is depicted under the header “Ontology” in https://platform.opentargets.org/disease/MONDO_0004975.

#### 2.1.2 Phase II: real-time knowledge retrieval

The chosen disease identifier from Phase I is used as a query for curated databases to retrieve relevant disease-associated knowledge in real-time. This is achieved by embedding the APIs of individual databases into our programmatic scripts and methods. The databases used as our source of knowledge and their details are described below.

##### 2.1.2.1 Open targets platform

The retrieval process begins by identifying human proteins and drugs associated with the disease in the OTP (Ochoa *et al.* 2021). The API for associated proteins results in a list of proteins with HUGO Gene Nomenclature Committee (HGNC) symbols, UniProt (Swiss-Prot) identifiers, Ensemble Gene identifiers and association scores. The latter is an OTP calculated score which intends to qualify the nature of the association and might serve as an attribute to filter the number of proteins that will be represented in the KG. The association score is influenced by a number of factors such as genetic associations, somatic mutations, drugs, RNA expression, and pathways of the proteins. The users are provided with a bar plot depicting the distribution of proteins based on the scores and can eventually choose a desired score threshold. Similarly, the API for protein target-associated-drugs results in a list of drugs along with annotations such as target protein within the context of the disease, preferred name, ChEMBL identifier, drug type, clinical trial identifier and drug development phase. Here again, the users are provided with the option to choose the drug development phase of the drugs. For instance, a user input of 2 will include all drugs annotated between clinical trial phase 2 and 4. Additionally, using the ChEMBL identifiers of drugs, associated warnings along with toxicity class and warning types and adverse events (i.e. side effects) along with counts and LogLR values are fetched from the OTP API.

##### 2.1.2.2 UniProt

Once a desired score is selected by the user, the UniProt identifiers of disease-associated proteins are used as input to the UniProt API. In this step, the workflow extracts protein-associated biological processes, molecular functions, and Reactome pathways (Milacic *et al.* 2024).

##### 2.1.2.3 ChEMBL

The ChEMBL identifiers of drugs associated with the diseases are used as input to the ChEMBL API to extract the knowledge of mechanism of action and active biological and functional assays (pChEMBL value > 6). In order to identify the assays, we used a combination of following filters: *Homo sapiens* as target organism, assay type biological or functional, confidence score of 9, i.e. direct single target protein and pChEMBL value greater than 6. The pChEBML value signifies the concentration, potency, or affinity of a chemical that elicits half the maximal response on a negative logarithmic scale. This enables comparison of aforementioned measurables which are usually represented using varying standard units including IC50, XC50, EC50, AC50, Ki, and Kd.

##### 2.1.2.4 GWAS Central

Next, the disease identifier is used as a query to GWAS central database through pandasGWAS (python package) (Cao *et al.* 2023) to collect genetic variants associated with human diseases. The database provides integrated data from different public repositories such as Single Nucleotide Polymorphism Database (dbSNP) (Sherry *et al.* 2001) and Database of Genomic Variants (DGV) (MacDonald *et al.* 2014), GWAS Catalog (Sollis *et al.* 2023) and published literature. The information of corresponding gene, variant identifier, its type (i.e. intron, intergenic, missense, synonymous, etc.) and disease-association scores are fetched from the database. Since, the number of SNPs for some diseases are extremely abundant, KGG represents only those SNPs that are detected within a gene sequence. The full list of SNP-gene associations with additional metadata is provided as a separate output file.

##### 2.1.2.5 Integrated interactions database (IID)

Lastly, the workflow can identify viral diseases affecting humans and integrate the biological activities of their proteins into the KG. In such cases, the taxonomy identifier of the virus is used as an input to the UniProt API to get its protein, to which we repeat the step explained above. Moreover, the workflow can consolidate virus mutants that cause the same disease. For example, AIDS (MONDO: 0012268) is caused by Human immunodeficiency virus 1 (Taxonomy identifier: 11676) and Human immunodeficiency virus 2 (Taxonomy identifier: 11709). Thus, the biological knowledge associated with both of these sub-species will be added to the KG.

#### 2.1.3 Phase III: KG compilation and generation

The retrieved knowledge from Phase II is stored as semantic triples (i.e. subject–predicate–object) using biological expression language (BEL), which are both human and computer-readable. The language enables systematic representation of biological and molecular interactions by enforcing usage of standard ontologies. The implementation was performed using the open-source PyBEL framework (Hoyt *et al.* 2018). It is a resource developed to help with triples formation, meta-data annotation, data parsing, validation, compilation, and visualization of KG. It also offers a wide range of functions to explore, query, and analyze KGs. The KGs can be exported to various standard formats such as json, csv, sql, graphml, and Neo4j, allowing comparison and integration with other KGs. In addition to the main goal of creating automated workflows for KGs, we have also developed workflows to compare disease-specific KGs, shared entities between KGs from other resources, retrieve PDB structures of proteins and explore the chemical space of drugs. The outputs of these methods are included in the results section.

## 3 Results

**Figure 2.**
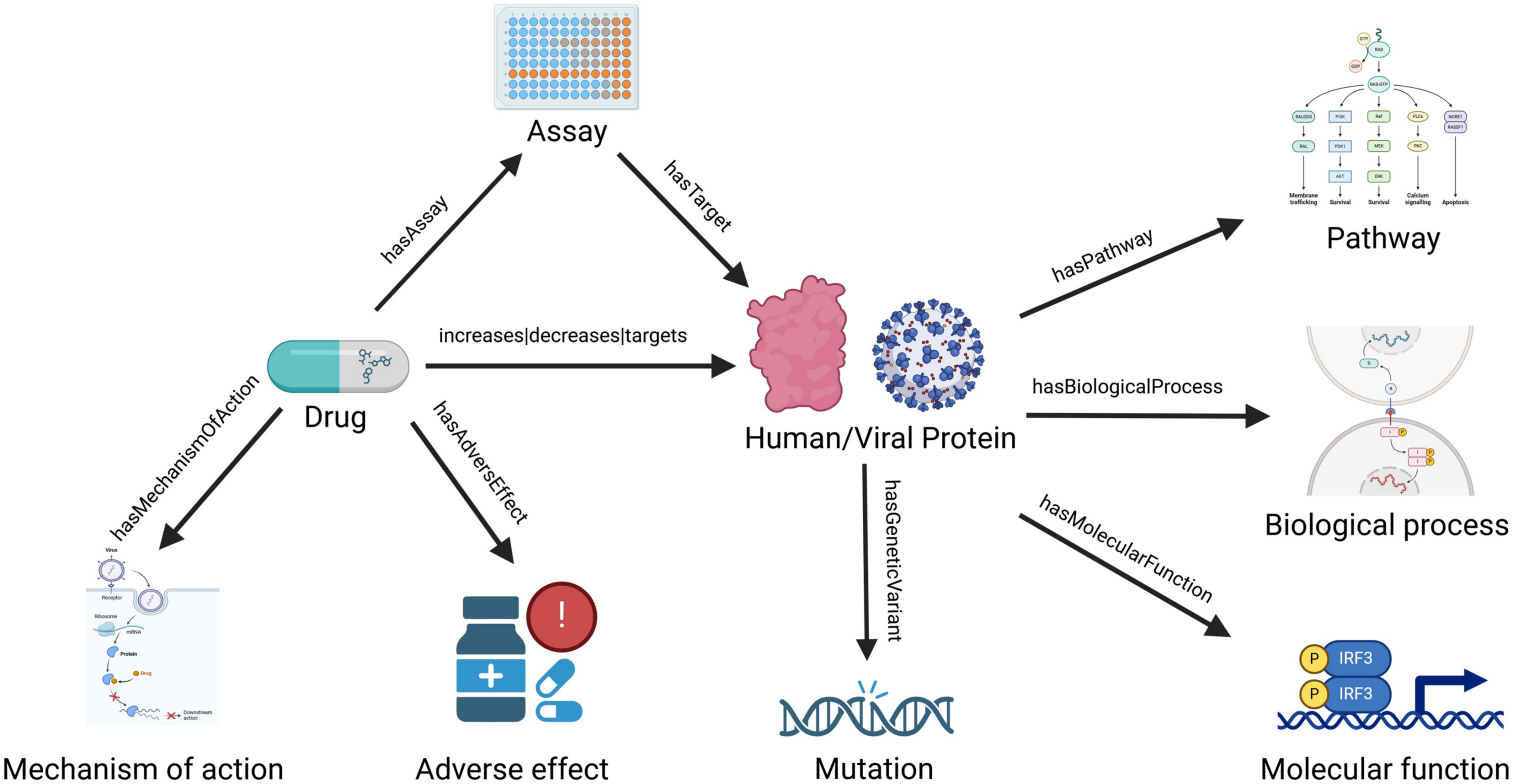
An illustration of KGG schema with represented entities and their relationships.

The KGG workflow yields a comprehensive graph constructed with systematically assembled genes and single nucleotide polymorphisms (SNPs), proteins and associated molecular processes and functions, chemicals and their target proteins, biological and functional assays, mechanism of actions and adverse events. A total of up to 9 different types of entities and 11 types of relationships are represented in a KG. The underlying schema of the KGG workflow is shown in Fig. 2 (https://biorender.com/tgb840q).

As shown in the figure, the relationship types are specific to the entities, which induce causality in the KG and represent a strong semantics. A special case of relationship between drugs and proteins exists as there are three possible types of relationships between them (i.e. “increases” or “decreases” or “targets”). The “increases” relationship type signifies that the activity of a drug can be a positive allosteric modulator, agonist, activator or partial activator (source ChEMBL). Whereas, “decreases” indicates that the drug exhibits either inhibitor, negative allosteric modulator, antagonist, or blocker activity. Lastly, “targets” relationship type is used represent miscellaneous drug activity such as modulator, opener and sequestering agent. This normalization was performed to avoid over-representing granularity of relationship types which can eventually lead to discrete and sparse connectivity of entities in the KG. However, the corresponding drug activities are available as annotations and can be conveniently used for specific queries, if needed.

The KG entities and relationships are also enriched with various metadata annotations, wherever applicable. For instance, drugs are annotated with preferred names, trade names and ChEMBL uniform resource locators (URLs). Similarly, gene ontology terms (biological processes and molecular functions) are annotated with their corresponding identifiers and URLs, as are proteins with UniProt URLs and druggability information. The relationships between drugs and assays are labeled with type of assays and pChEMBL values whereas relationships between proteins and reactome pathways are labeled with reactome identifiers. These annotations can be used in filtering and querying the graph.

The depiction of these entities in the KG is done by using standard ontologies or identifiers. For instance, human proteins are represented with HUGO names and are further annotated with UniProt identifiers. Likewise, chemicals and chemical assays are represented with ChEMBL identifiers. In addition, the process of creating a KG produces several intermediate files, all of which are saved as individual files for the convenience of the users. The KGG outputs from this study and their additional downstream analyses are explained with following use cases.

### 3.1 Use case 1: comorbidity between COVID-19 and Alzheimer’s disease

**Figure 3.**
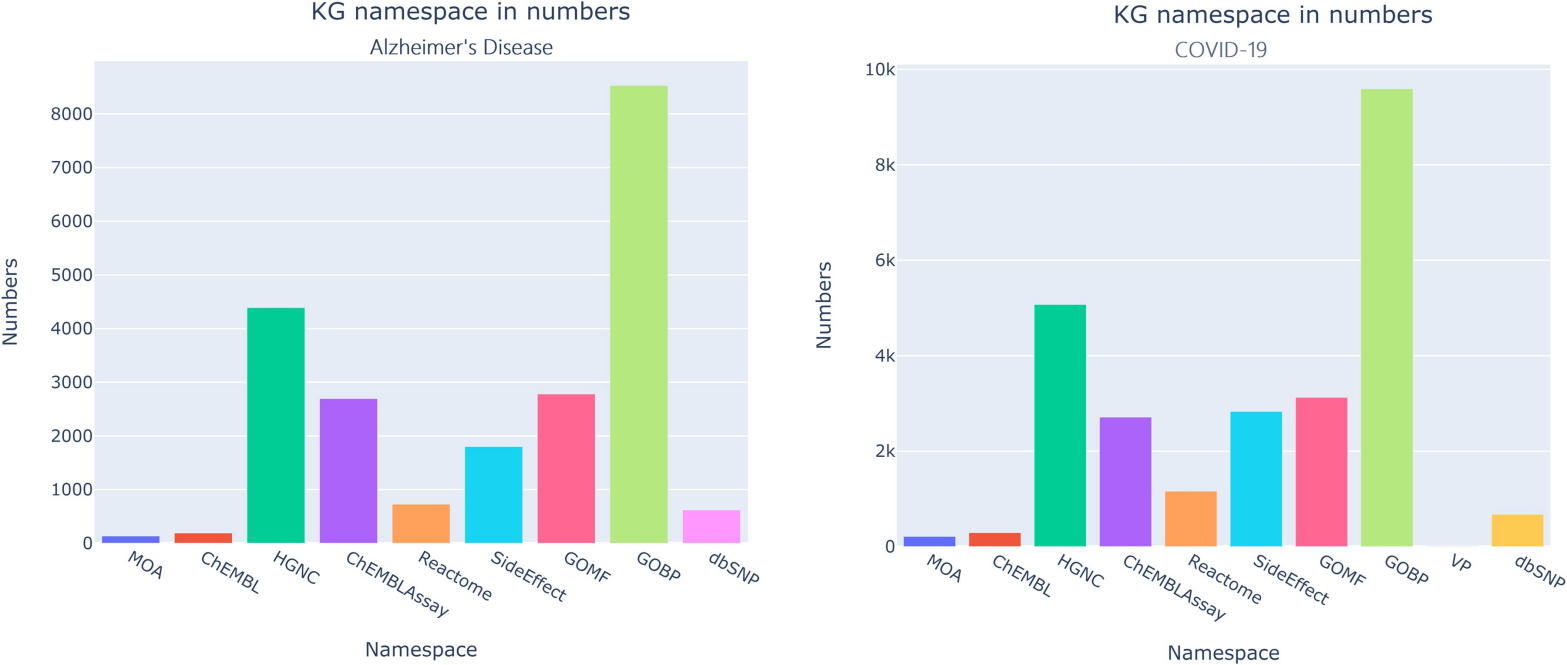
Bar plots showing entities and their numbers in COVID-19 KG (left) and AD KG (right).

Amidst the speculation of possible of link between COVID-19 (MONDO: 0100096) and AD due to the findings such as over-expression of a major COVID-19 related protein (i.e., ACE2) in brains of AD patients (Lim *et al.* 2020) and AD risk factor (i.e., APOE e4 allele) facilitating SARS-CoV-2 virus infiltration (Yin *et al.* 2021, Chen *et al.* 2023), we used our workflow to generate new insights about these diseases. We firstly created individual KGs for both the diseases and afterwards identified the shared number of proteins, drugs, biological processes, and SNPs between these diseases. The COVID-19 KG consisted of 25 701 entities and 206 787 triples whereas the AD KG consisted of 21 880 entities and 149 515 triples (Fig. 3).

**Figure 4.**
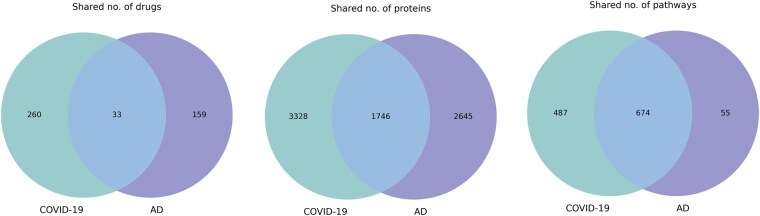
Venn-diagrams showing shared entities between COVID-19 and AD.

We found that a total number of 1746 proteins, 33 chemicals, 674 pathways and 2 SNPs were shared between them (Fig. 4). Interestingly, out of the two SNPs, we identified a novel SNP (i.e., rs11065822), which is associated with the CUX2 gene, to be linked to both the diseases. Whereas, rs429358, which is linked to APOE, has been previously reported as shared genetic between COVID-19 and AD (Matveeva *et al.* 2023). Next, we merged both the KGs to generate a comprehensive KG consisting of 33 052 entities and 274 925 triples.

### 3.2 Use case 2: Depression KG

#### 3.2.1 KG in numbers

The KGG-generated Depression KG (MONDO: 0002009) is composed of 15 047 entities and 89 531 triples. Among the entities are a total of 2429 proteins, 108 drugs, 6875 biological processes, 1962 molecular functions, 179 reactome pathways, 1136 genetic variants, 889 assays, 1425 adverse effects, and 44 mechanism of actions. A bar plot showing the distribution of these entities is provided in Fig. 1, available as supplementary data at *Bioinformatics* online.

#### 3.2.2 KG comparison with Depression models from other sources

The comparison between the Depression Knowledge Graph (KG) built using KGG and the text-mining approach (Kairntech Sherpa) was conducted for shared proteins, chemicals, and biological processes. Sherpa, a web-based, user-friendly tool, is designed for tasks such as relation extraction and information retrieval (Geißler 2020). Previously, this tool has been effectively and efficiently used to extract BEL triples from textual corpora focused on the modulation of MAPT (tau) phosphorylation (Babaiha *et al.* 2023, 2024). The text processing within Sherpa follows several key stages, including tokenization, named-entity recognition (NER), and named-entity normalization (NED). In Sherpa, NER and NED are combined into a single framework using a machine-learning method called Entity Fishing (https://github.com/kermitt2/entity-fishing). Following this, a specialized model is trained and integrated into the NLP pipeline to detect specific features of entities, such as protein modifications. The final step involves using OpenNRE, a neural network-based relation extraction (RE) tool to determine and classify the relationships between entities (https://github.com/thunlp/OpenNRE).

To build the text-mined KG for depression, we searched for publications annotated with the Depression MeSH term in PubMed. A total of 22 830 abstracts thus identified were programmatically retrieved using the Metapub Python library (https://pypi.org/project/metapub), which interacts with NCBI’s Eutils (Entrez Programming Utilities). The Sherpa pipeline was then applied to these chunks of text, extracting all relevant BEL triples. The resulting text-mined KG consisted of 3701 entities and 8919 triples, where the numbers of proteins, chemicals and biological processes were 752, 464, and 247, respectively.

Since the proteins and biological processes were represented with HGNC symbols and Gene Ontology respectively, they were readily comparable with our KG. However, the chemicals had to be normalized to ChEMBL identifiers as they were originally represented with CHEBI identifiers. After performing the normalization, we found that a total number of 462 proteins, 60 chemicals, and 42 biological processes were shared between the two KGs (Fig. 2, available as supplementary data at *Bioinformatics* online).

#### 3.2.3 Identification of PDB structures

**Figure 5.**
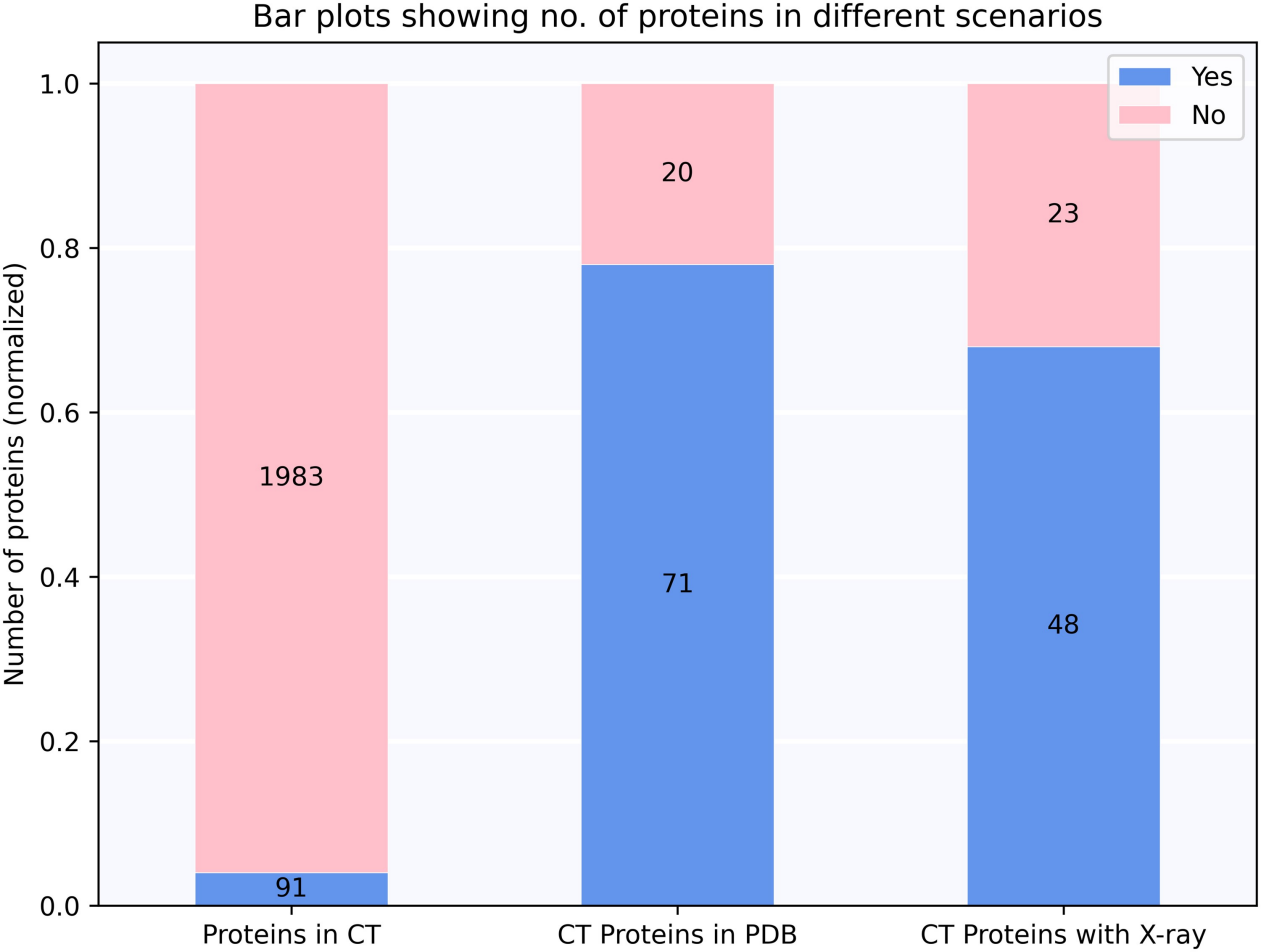
Stacked bar plots showing number of Depression-proteins in clinical trials and PDB.

We took a step forward to identify PDB identifiers for Depression-specific proteins as structural information is key to drug discovery. We first filtered out on proteins which have been targeted by Depression drugs in various clinical trial phases. This resulted in 109 drugs with 91 target proteins. Next, we used the UniProt identifiers to identify corresponding PDB structures including known X-ray structures. A total of 71 proteins were reported to have PDB structures among which ESR1 (P03372) had the highest number of structures, i.e. 439 PDB and 435 X-ray crystallography structures. Out of 71 proteins, 48 proteins had at least one X-ray structure whereas structures of 20 proteins out of 91 are yet to be discovered (Fig. 5). The table for Fig. 5 is provided as Table 1, available as supplementary data at *Bioinformatics* online.

### 3.3 Use case 3: Parkinson disease KG (MONDO: 0005180)

#### 3.3.1 KG in numbers

The PD KG (MONDO: 0005180) is composed of 20 686 entities and 170 754 triples. Among the entities are a total of 4245 proteins, 166 drugs, 9379 biological processes, 3041 molecular functions, 413 reactome pathways, 269 variants, 986 assays, 2079 adverse effects, and 108 mechanisms of action. A bar plot showing the distribution of these entities is provided in Fig. 3, available as supplementary data at *Bioinformatics* online.

#### 3.3.2 KG comparison with manually curated Parkinson disease map

The PD map, created with Systems Biology Graphical Notation (SBGN), depicts underlying molecular interactions, dysfunctional biological processes and pathways of PD pathogenesis. This knowledge is manually curated from 1587 scientific publications and enriched with additional metadata annotations from several bioinformatic databases (Fujita *et al.* 2014). The map is hosted using the MINERVA Platform (https://minerva.pages.uni.lu) and offers intuitive and user-friendly tools for analyses such as overlaying experimental data and identification of drug targets and chemical interactions (Gawron *et al.* 2016). Upon comparing the PD map with KGG-generated KG we found that a total of 3 drugs, 654 proteins, and 188 biological processes were shared between them (Fig. 4, available as supplementary data at *Bioinformatics* online).

#### 3.3.3 Exploration of chemical space of PD drugs in clinical trials

**Figure 6.**
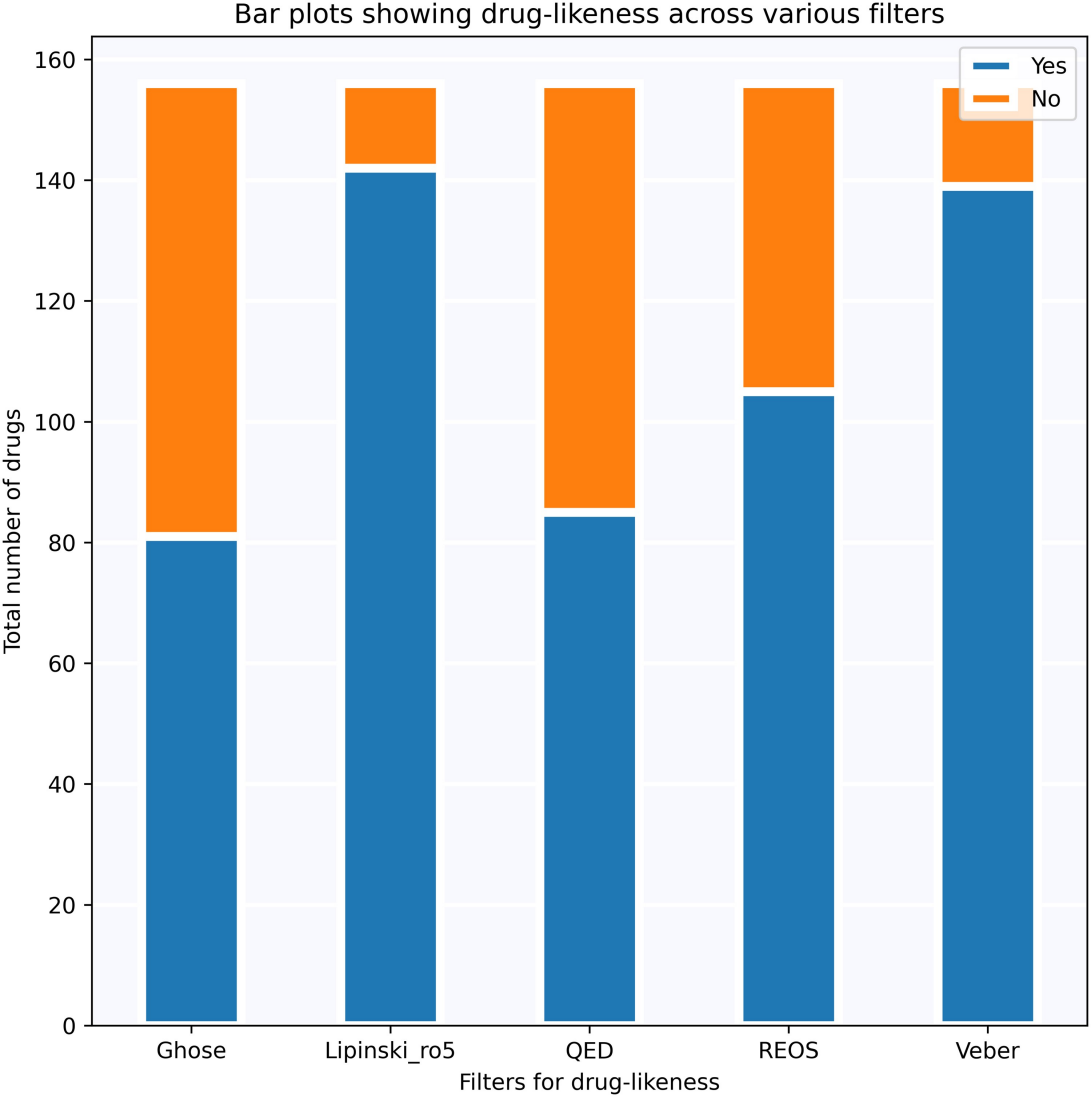
Drug-likeness profiles of PD drugs using various filters.

**Figure 7.**
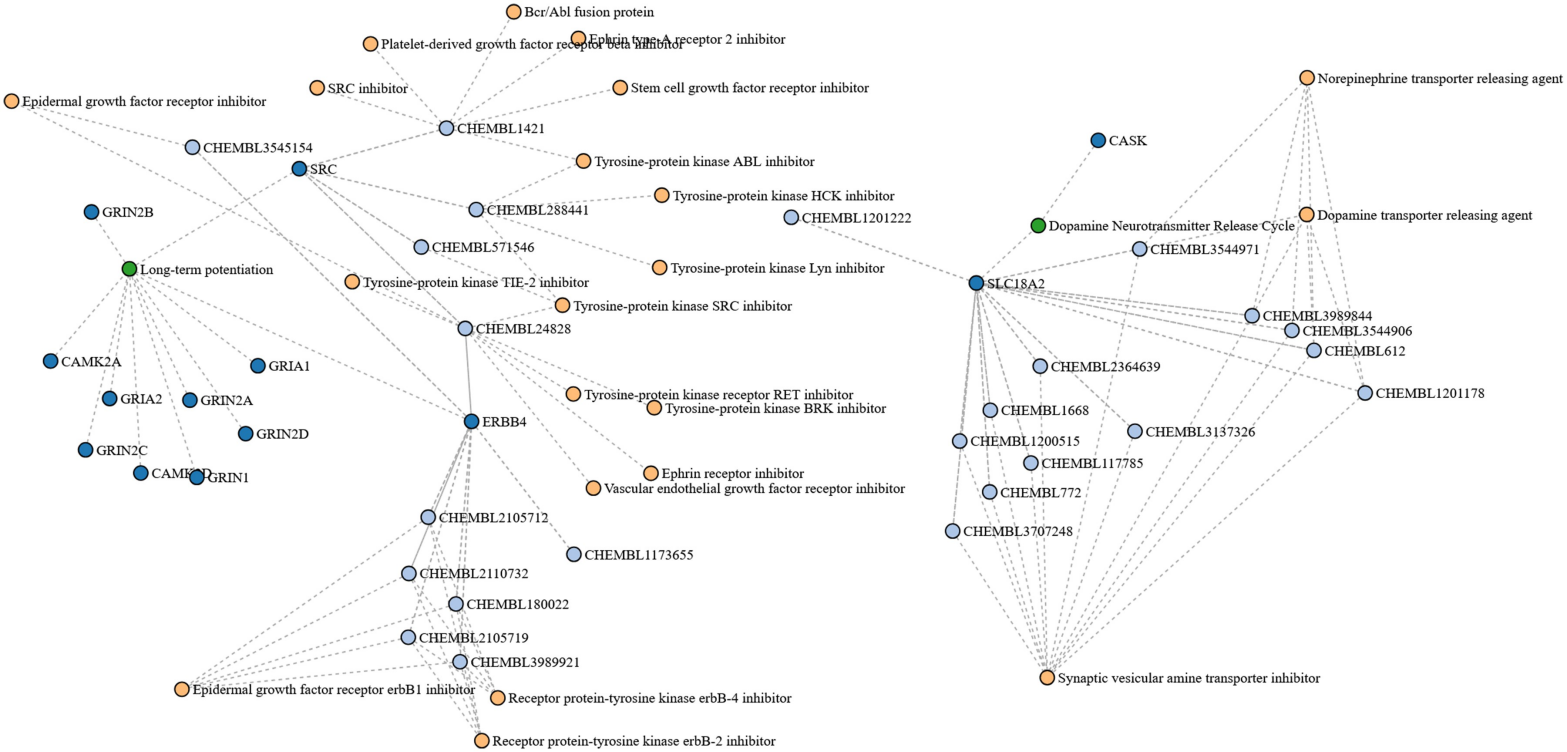
A sub-graph depicting druggable proteins, their pathways and repurposing drug candidates in Parkinson Disease (PD).

A total of 121 (12.2%), 400 (40.2%), 134 (13.5%), and 340 (34.2%) drugs were in clinical trial phase I, II, III, and IV respectively. A pie-chart summarizing proportion of PD drugs in different clinical trial phases is shown in Fig. 5, available as supplementary data at *Bioinformatics* online. As the same drug can be part of multiple clinical trials, the number of drugs is not unique in this case. The distribution of drugs based on their types (e.g. small molecule, antibody, etc.) is shown in Fig. 6, available as supplementary data at *Bioinformatics* online. Next, we conducted a comprehensive drug-likeness evaluation of PD drugs using five different methods, i.e. Ghose, Lipinski rule of 5, quantitative estimation of drug-likeness (QED), rapid elimination of swill (REOS), and Veber (Fig. 6). These methods offer *in silico* insights into the oral bioavailability of drugs by calculating various physicochemical properties from their SMILES representations. To achieve this, we filtered out drugs of types unknown, protein and antibody as they do not have any SMILES. Among these drugs, 81, 142, 85, 105, and 139 drugs met the criteria for Ghose, Lipinski rule of 5, QED, REOS, and Veber, respectively. Notably, only 46 drugs passed all five filters, with 6, 19, 9, and 12 drugs in clinical trial phases I, II, III, and IV, respectively. Conversely, nine drugs did not pass any filters, with 1, 3, 1, and 4 drugs in clinical trial phases I, II, III, and IV, respectively. Detailed summaries of physicochemical properties of the drugs, their evaluation with the drug-likeness methods, and the highest clinical trial phase are available in Table 2, available as supplementary data at *Bioinformatics* online. An interactive parallel coordinates plot for this analysis is also provided in Fig. 7, available as supplementary data at *Bioinformatics* online.

#### 3.3.4 Identification of putative new targets and repurposing drug candidates in PD

The PD KG was then subjected to neighborhood-based analysis for identifying putative new drug targets in PD by contextualizing the knowledge of proteins in clinical trials and associated pathways. We firstly created a sub-graph of the KG by filtering all proteins and their corresponding pathways. Afterwards, we retained 211 proteins targeted by 169 drugs in clinical trial phases, resulting in a total of 293 pathways. Next, the neighborhood around these pathways was expanded by including proteins that had not been previously considered as drug targets in PD, followed by the removal of those labeled as “No” for “Druggability” based on the metadata annotation from the OTP. From the resulting sub-graph with a total of 733 proteins, we identified that G alpha (s), (i), and (q) signaling events from the G protein-coupled receptor (GPCR) downstream signaling were the top three represented pathways with 22, 18, and 12 respective proteins used as drug targets in clinical trials. For these pathways, a total of 32, 49, and 43 druggable proteins were identified in the sub-graph.

As a use case in this study, we chose to identify repurposing drug candidates for proteins involved in long-term potentiation (7 proteins in clinical trials and 4 druggable proteins i.e., CAMK2A, CAMK2D, ERBB4, and SRC from the KG) and dopamine neurotransmitter release cycle (none in clinical trial and 2 druggable proteins i.e., CASK and SLC18A2 from the KG). These pathways are known to be important for synaptic plasticity and cognition (Cooke and Bliss 2006, Zhou *et al.* 2023). We used the six druggable proteins to identify associated drugs from clinical trials of other diseases. After filtering for drugs in phase III and IV to ensure their efficacy, safety and higher clinical value, a total of 8, 13, and 4 repurposing drug candidates were found for ERBB4, SLC18A2, and SRC, respectively, whereas no drugs were found for CAMK2A, CAMK2D and CASK (Fig. 7). Additionally, in another perspective, we also explored the highest number of pathways associated with each druggable proteins which are not yet in clinical trials. We identified PIK3R1 (24), JAK2 (18), MAPK1 (16), MAPK3 (15), CAMK2A (14), CSNK1A1 (14) and SRC (14) as proteins with highest number of associated pathways. To these, we found 17, 4, and 4 drug candidates for JAK2, PIK3R1, and SRC, respectively in phase III and IV. The results of these analyses are available in Table 3, available as supplementary data at *Bioinformatics* online.

## 4 Discussion

In this work, we introduce a fully automated workflow for generating KGs by integrating APIs of several curated databases. To our knowledge, KGG is the first workflow that enables users to create KGs by fetching data in real-time. With our approach, we believe that we have taken a significant step toward fast and rapid generation of KGs as compared to conventional methods that are time consuming and require a lot of manual effort. This generalized approach has been revamped on our previous works which focused on improving COVID-19 data findability and interoperability (Ohmann *et al.* 2023), proposing appropriate frameworks for life science research data (Ohmann *et al.* 2023) and preparing for possible threats of infectious diseases and pandemics (Karki *et al.* 2023). Overall, KGG is a pragmatic demonstration of Findable, Accessible, Interoperable, and Reusable (FAIR) principles focusing especially on the reusability aspect. Nevertheless, the major credit and thanks are owed to curated databases who have put enormous efforts in providing high-quality data to the scientific community.

The current version of KGG workflow incorporates up to 9 different types of entities and can be expanded to represent new entities and metadata annotations. For instance, the OpenBEL framework efficiently supports delineation of post-translational modifications of proteins with appropriate syntax and structures. Likewise, the impact of functional effects of SNPs in the amino acid substitutions in proteins can be conveniently encoded using PolyPhen-2 database (Adzhubei *et al.* 2013). In fact, the use of standard ontologies and vocabularies for entity representation enables interoperability and provisions seamless integration of untapped databases and resources. With regards to annotations, we have added entity and relationship-level metadata including preferred names and ChEMBL URLs for drugs, UniProt identifiers and druggablility information for proteins and assay type and pChEMBL values for chemical–assay relationships. The annotations can be further enriched with user-specific data such as genomics/proteomics, drug class and its physicochemical properties. Our future work and codebase releases will focus in expandability of the KGG workflow whereby we will broaden the spectrum of biochemical nodes and their relationships with appropriate metadata annotations followed by tools for knowledge graph applications.

Our use cases have compared KGG-derived KGs with KGs from other resources for identification of commonalities and discrepancies between them. One important point worth mentioning is that KGs are created with different scopes and objectives, therefore discrepancies in KGs are inevitable. Our analysis showed that our workflow uniquely captured certain entities such as ChEMBL assays, SNPs, mechanisms of action and side effects. It is also evident from the numbers of entities, their types and triples that the coverage of KGG is much larger than the other resources. This is because our workflow incorporates up-to-date high-quality knowledge from curated databases which exceeds the number of overall scientific publications (Depression and PD KG were built with 22 830 text-mined abstracts and 1587 full text articles, respectively). However, the text-mined KG consisted of additional types of chemicals including hormones, chemical elements and drug type/class which was not the case in our KG as KGG is a drug-centric workflow. Nevertheless, in the context of drugs, our KG captured all 108 Depression-specific drugs which was not the case with the text-mined KG. The text-mined KG also included other Depression-related diseases which was not the case in our KG. Likewise, the PD map was able to capture only 32 drugs as compared to 169 drugs in our KG which can be directly attributed to the relatively lower number of curated articles and the focus to capture molecular mechanisms. On the other hand, the PD map was highly enriched with protein-protein interactions (PPIs) specific to PD which were missing in our KG. All in all, it can be concluded that KGs from different frameworks and resources can be integrated to create comprehensive KGs. We did not consider doing it as it was outside the scope of our work and required more effort and time. Nevertheless, the integration of KGG-derived COVID-19 KG and AD KG to create a comprehensive KG is the showcase of such possibility and was swiftly done as they were created with the same framework. The PD KG use case further highlights the importance of disease-specific KGs as we were able to demonstrate how the knowledge of proteins in clinical trial phases along with their pathways can be used to identify putative new druggable proteins. This pathway-centric approach enabled us to prioritize proteins by providing insights of corresponding pathways and subsequently retrieve repurposing drug candidates from advanced clinical trial phases of other diseases. Specifically, it was interesting to discover SLC18A2 as a new target and its drugs that regulate the dopamine release cycle by functioning as neurotransmitter releasing agents. We could support this by finding previous studies which have implicated SLC18A2 as a therapeutic target in PD (Lohr *et al.* 2014, Lohr and Miller 2014). The utility of KG in exploration of all possible new targets, their prioritization and identification of repurposing candidates will be considered in a systematic manner in our future work.

One of the major weaknesses of the KGG workflow is reflected in “not-well-studied” diseases which yield sparse or non-connected KGs. This is directly affected by our dependence on curated databases which are our primary sources of knowledge. Also, the KGG is limited to represent top-level concepts of pathways as the workflow misses out on detailed mechanistic events of pathophysiological phenomena captured in PD map and other manually curated KGs. This highlights the importance of manually curated KGs but at the same time provides the rationale why such KGs are always confined to limited articles. Our workflow currently also does not incorporate disease-specific PPIs which are considered to be unique for each disease as disease mechanisms are driven by dysregulated PPIs. With these considerations, we see the potential of integrating local experimental data, text-mining workflows and LLMs to enrich the KG for its comprehensiveness. As already discussed before, it can be ensured that these computer-aided methods will have even a bigger impact in the knowledge extraction and retrieval process in the near future.

The KGG is developed for a broad spectrum of researchers and scientists, especially for those who are involved into pre-clinical drug discovery, understanding disease mechanisms/comorbidity, and drug repurposing. It comes with a user-friendly and interactive interface [i.e. dashboard implementation in Streamlit (https://fraunhofer-itmp-ds-toolkit.serve.scilifelab.se/KGG) and Visual Studio Code/Jupyter Notebook for programmatic use] to take inputs from users and run the underlying scripts and methods. It is designed to enable researchers with minimal knowledge of programming to generate KGs at convenience. The computer scientists can, however, make maximum advantage of the workflow by modifying the scripts according to their needs. One specific use case of KGG is to get the knowledge of important proteins and their functions within the context of any possible outbreak of infectious diseases. Moreover, researchers can also quickly identify drugs in different clinical trial phases of those diseases. Similarly, KGG can also help researchers to design their experiments as it incorporates knowledge of previously reported active assays and corresponding targets. In addition to the main goal of creating automated workflows for KGs, we have also developed workflows to compare disease-specific KGs, shared entities between KGs from other resources, retrieve PDB structures of proteins and explore drug-likeness of chemicals. Since KGs have a wide range of applications, they will be of interest to bioinformaticians and chemoinformaticians for defining their own use cases and deploying machine-learning methods for advanced analyses.

## Supplementary Material

btaf383_Supplementary_Data

## Data Availability

The scripts and codes associated with this study are available in GitHub at https://github.com/Fraunhofer-ITMP/kgg.
