# Supplementary figures and images for: KGG: a fully automated workflow for creating disease-specific knowledge graphs
Source: Bioinformatics. 2025 Jun 28;41(7):btaf383. doi: 10.1093/bioinformatics/btaf383 (PMC12270262; doi:10.1093/bioinformatics/btaf383)

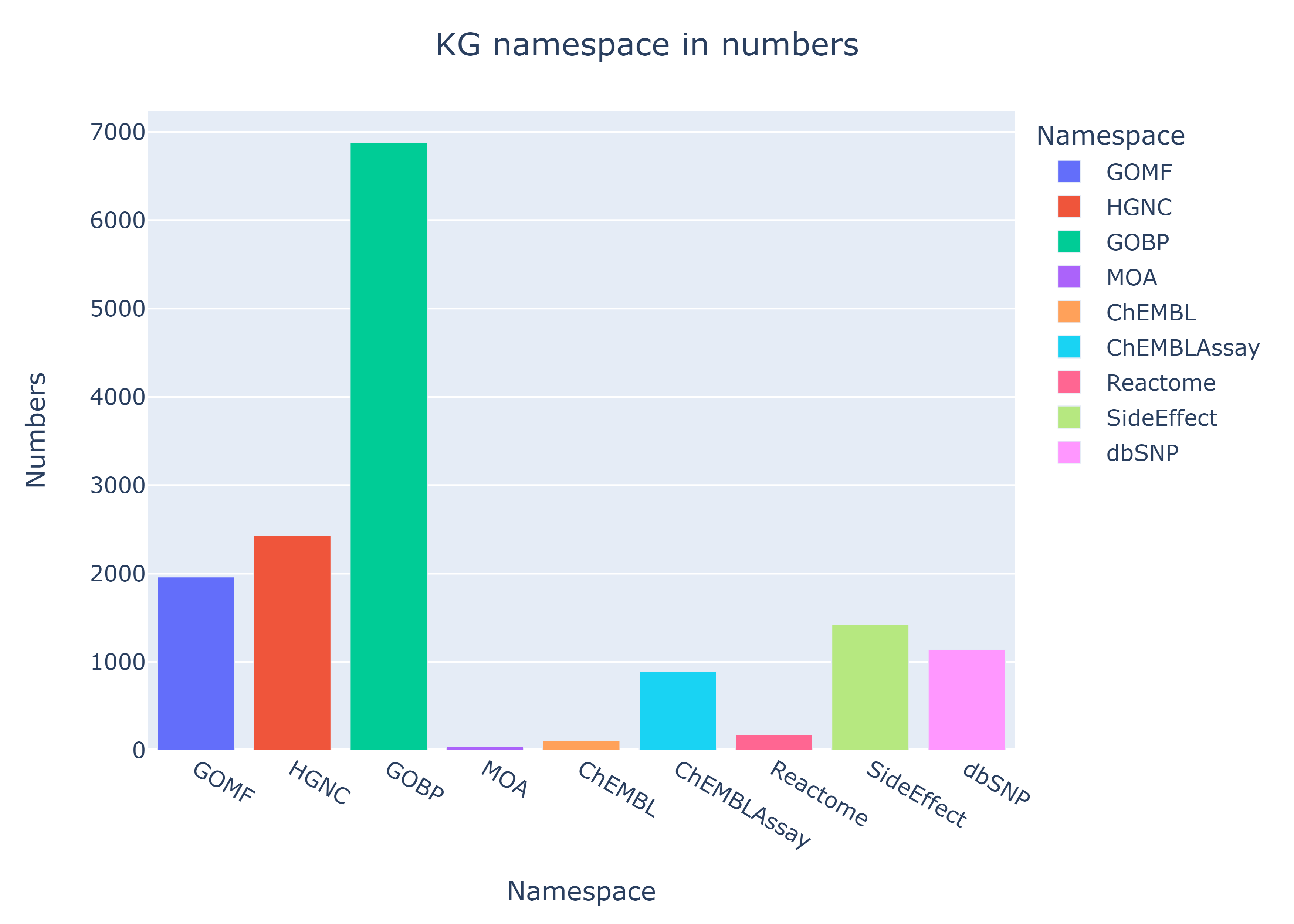

Supplement: btaf383_Supplementary_Data [file btaf383_supplementary_data.zip › Supplementary Figure 1.png]

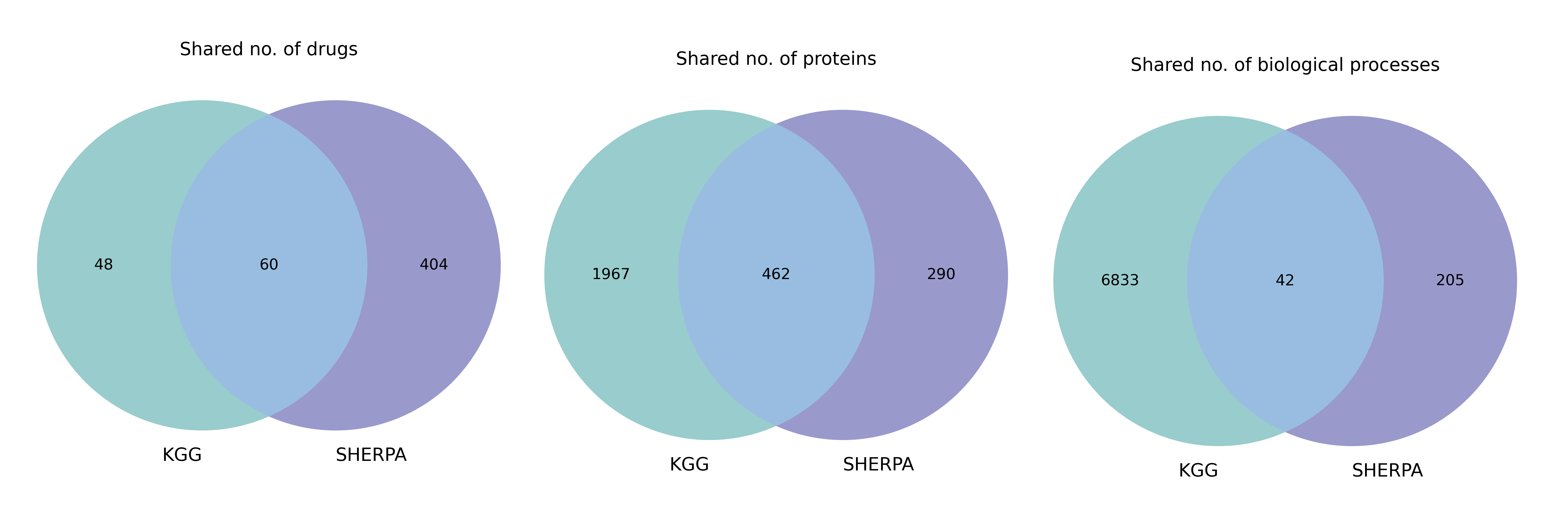

Supplement: btaf383_Supplementary_Data [file btaf383_supplementary_data.zip › Supplementary Figure 2.png]

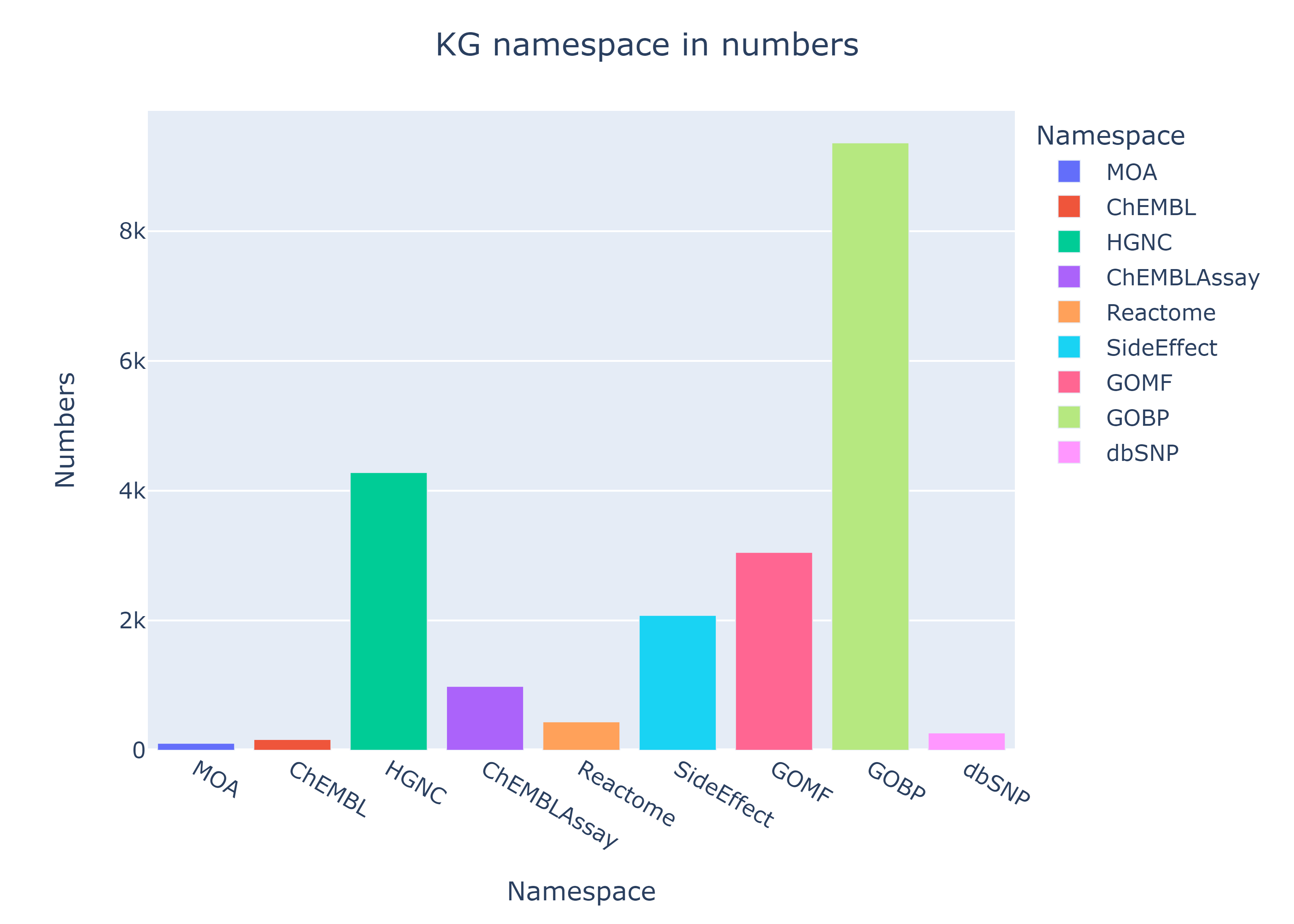

Supplement: btaf383_Supplementary_Data [file btaf383_supplementary_data.zip › Supplementary Figure 3.png]

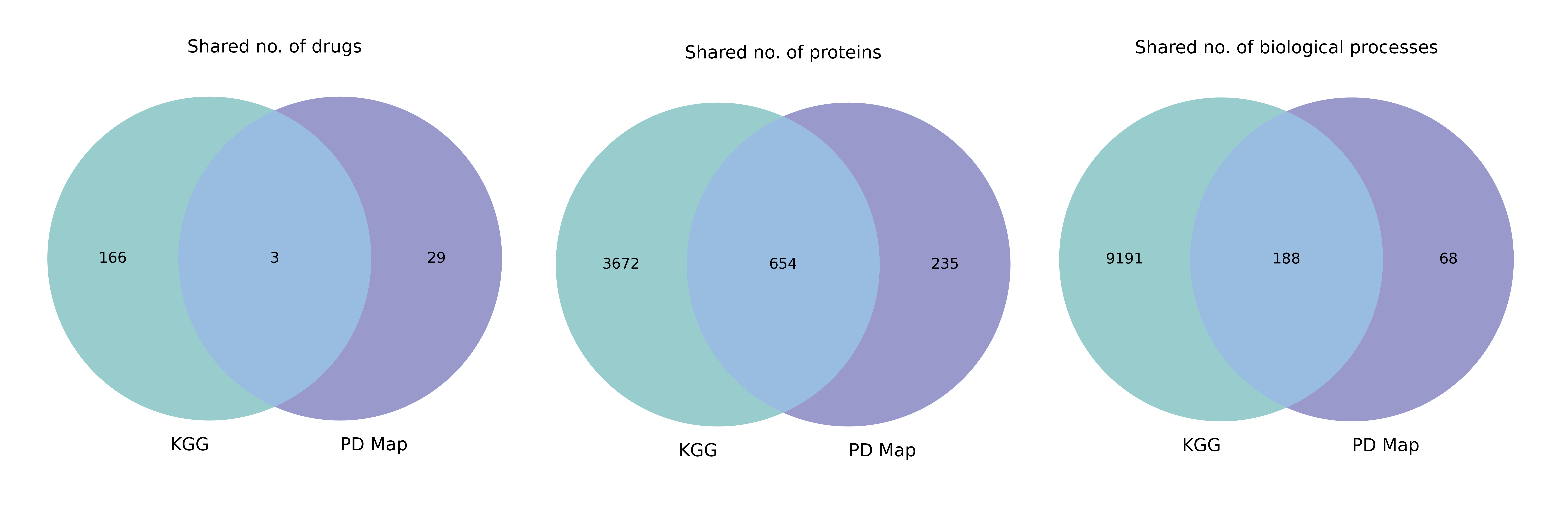

Supplement: btaf383_Supplementary_Data [file btaf383_supplementary_data.zip › Supplementary Figure 4.png]

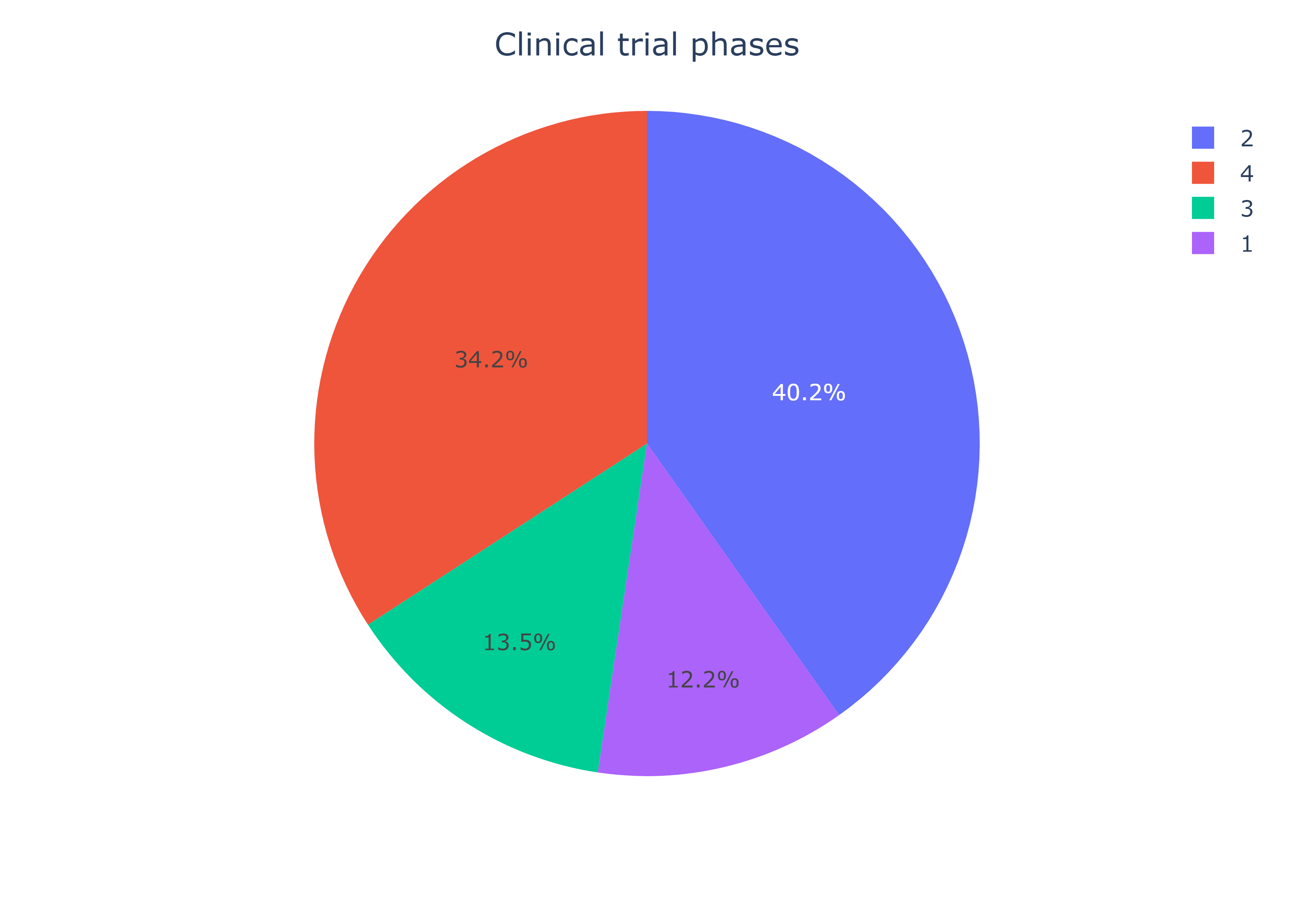

Supplement: btaf383_Supplementary_Data [file btaf383_supplementary_data.zip › Supplementary Figure 5.png]

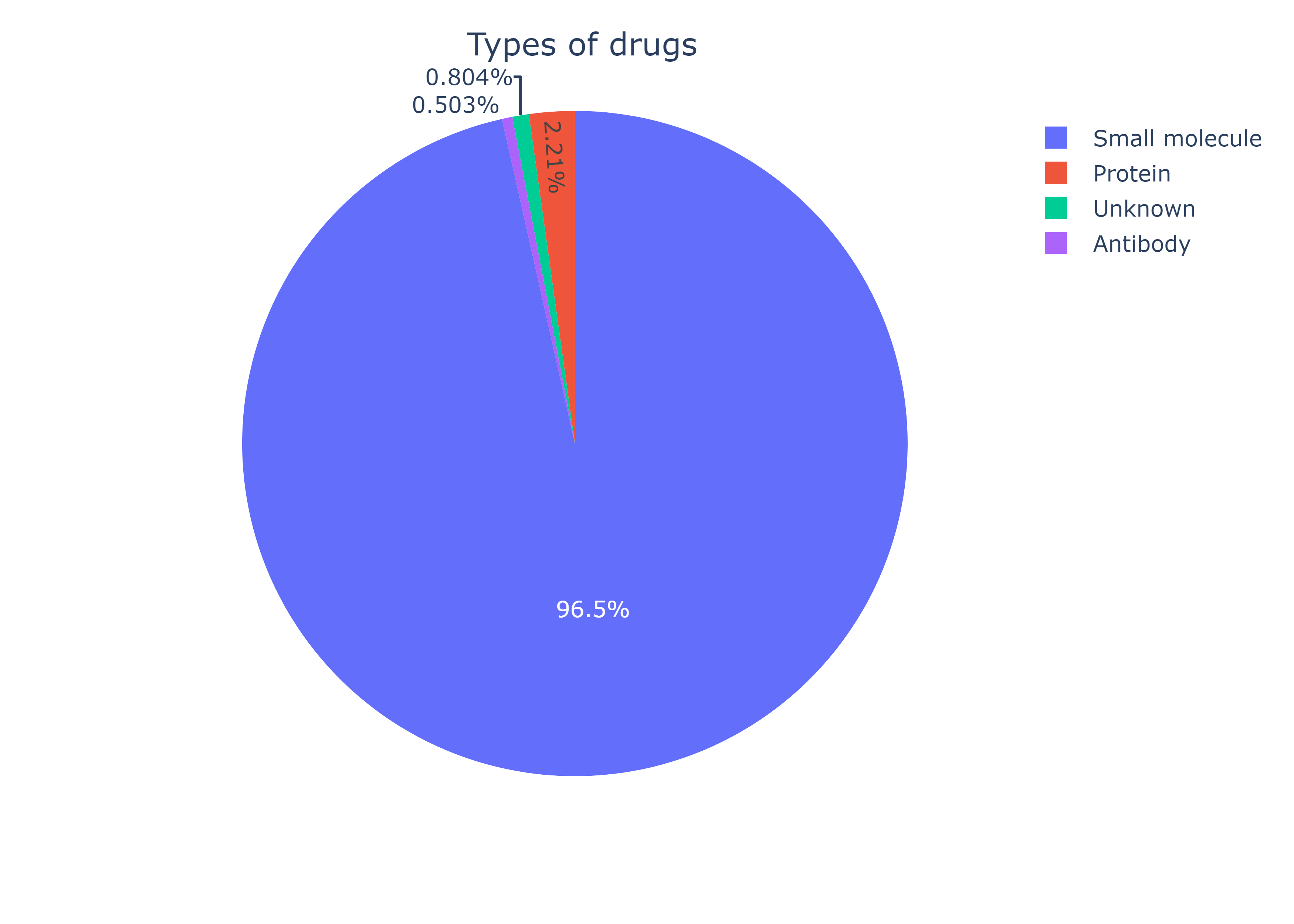

Supplement: btaf383_Supplementary_Data [file btaf383_supplementary_data.zip › Supplementary Figure 6.png]
